# Retention of Pro-Vitamin A Content in Products from New Biofortified Cassava Varieties

**DOI:** 10.3390/foods8050177

**Published:** 2019-05-24

**Authors:** Toluwalope Emmanuel Eyinla, Busie Maziya-Dixon, Oladeji Emmanuel Alamu, Rasaki Ajani Sanusi

**Affiliations:** 1 Food and Nutrition Sciences Laboratory, International Institute of Tropical Agriculture, PMB 5230, Ibadan, Oyo State, Nigeria; t.eyinla@cgiar.org; 2 Department of Human Nutrition, College of Medicine, University of Ibadan, PO Box 22133, Ibadan Oyo State, Nigeria; sanusiadegoke2003@gmail.com; 3 Food and Nutrition Sciences Laboratory, International Institute of Tropical Agriculture, Southern Africa Research and Administration Hub (SARAH) Campus PO Box 310142, Chelstone, Lusaka 10101, Zambia; o.alamu@cgiar.org

**Keywords:** Cassava, gari, retention, beta-carotene, vitamin A intake

## Abstract

Plant breeding efforts in sub-Saharan Africa (SSA) have produced biofortified cassava with high carotenoid content to address vitamin A deficiencies (VAD). Since carotenoids in foods are easily depleted during processing, the retention of β-carotene in some newly released cassava varieties is under query. From four of these new varieties, two commonly consumed products (gari and its dough) were processed according to standard methods. Retention of β-carotene was then probed after applying fermentation periods of a day and three days. The possible contribution of the products to Vitamin A intake in children, adolescents, and women was also assessed. The concentration of β-carotene in fresh Cassava roots ranged from 5.32 to 7.81 µg/g. The percentage retention ranged from 14.4 to 29.3% and 10 to 21.7% in gari fermented for one and three days respectively. The impact of varietal difference and length of fermentation was significant on retention in the intermediate and final products (*p* < 0.001). When compared with dietary intake data, cooking biofortified gari into its dough reduced Vitamin A intake in most varieties. We conclude that processing Cassava into gari (especially its dough) could hinder the retention of β-carotene however some varieties have retention advantage over others irrespective of the initial concentration in their fresh roots.

## 1. Introduction

Vitamin A deficiency (VAD) is still a prevailing public health challenge in many sub-Saharan countries [1]. While several interventions have attempted to reduce this burden, few have provided the promise of sustainable impact on a large scale when compared with biofortification of crops [2,3]. The main advantage of biofortification rests on the selection of crops which are usually staples of selected populations, thus increasing their adaptability [4]. This is true of a crop like cassava in Africa where it is a widely used and consumed staple especially in underdeveloped populations [5,6,7,8,9]. Another advantage of biofortification is that unlike other interventions that may seek to deliver a high instant dosage of micronutrient through food supplementation/fortification, biofortifying staples will consistently contribute to daily micronutrient intake, so far, the crop is consumed [10].

Thus cassava, which is a chief source of dietary carbohydrate in local diets, when biofortified with increased levels of carotenoids, can now offer other nutritional benefits, such as contributing to improved functioning of visual and immune systems [11], and possible inhibition of carcinogenic pathways [12]. In Nigeria, where a strong breeding effort exists, there have been releases of high pro-vitamin A content cassava varieties since 2011 [13,14]. These varieties also popularly referred to as “yellow cassava” which are being promoted in various communities and are gaining momentum across the country [15]. These successes have diffused into neighboring countries within sub-Saharan Africa (SSA) with a release of similar varieties to combat VAD in burdened populations.

However, retention of carotenoids is still a challenge during the processing of fresh yellow cassava roots into commonly consumed products mainly due to the sensitive nature of carotenoids to light, heat and physical handling [16,17]. Thus, the retention of total carotenoids is usually dependent on the prevalent processing method and the variety being used. The former being difficult to control especially in large scale processing which is common in SSA

While previous studies have highlighted the retention of total carotenoids in cassava products [13,17,18] few studies have specifically examined the effects of processing on β-carotene—the principal carotenoid in biofortified cassava [19,20,21]. Also, studies examining β-carotene retention at each step of processing of cassava into commonly consumed local products are scarce [17,20,21]. Another justification for this probe is that, even though there is a report on retention in fermented dough made from biofortified Cassava [20], no study has evaluated β-carotene retention in high carotenoid content varieties, which were most recently released in 2014, especially when processed into commonly consumed products (gari and its dough). Gari is a roasted granule obtained through processing (fermenting, grating, dewatering, and frying) of fresh cassava roots, and its dough “eba” is obtained by cooking gari in hot water to the constant dough. These products constitute a major part of the dietary intake of cassava products in Nigeria and SSA [22,23].

In this study, β-carotene concentrations and their retention, in gari and its dough “eba” were studied under two fermentation periods. The study also evaluated the possible contribution of these products to Vitamin A intake by comparing β-carotene concentrations in yellow varieties with dietary data of analogous products from the white cassava variety.

## 2. Materials and Methods

### 2.1. Experimental Design

A laboratory experimental design was used to evaluate the concentrations and retention of total β-carotene in four varieties of biofortified cassava and their products. Comparison of laboratory results with dietary data of cassava products (white variety) was used to estimate the contributions of yellow cassava products to the Recommended Dietary Allowance (RDA) of vitamin A in selected respondents.

### 2.2. Harvesting and Processing

**Figure 1:**
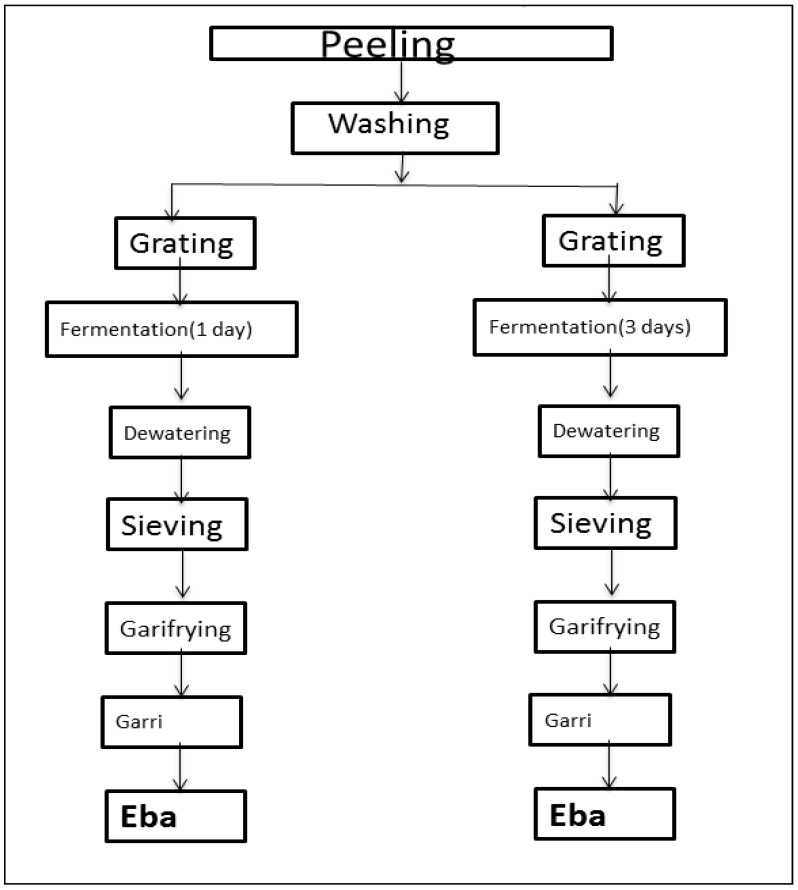
Schematic diagram of steps involved in the processing of fresh raw cassava roots into Garri and Eba using two fermentation periods [24,25,26,27].

Matured roots (aged between 11 and 12 months) of four recently released yellow-fleshed cassava varieties—TMS 0593, TMS 0539, NR 0220, and TMS 1371—were harvested from the research farm of International Institute of Tropical Agriculture (IITA), Ibadan, Nigeria. Large and small roots were selected in a proportional manner across all varieties. The total weight ranged between 5 kg and 7 kg. Damaged roots were sorted out. The roots were then peeled, washed, and processed into commonly consumed cassava products—gari and its dough. Two processing batches were carried out by fermenting the grated mash for one day and three days as explained in Figure 1. The same experimental conditions were applied uniformly across both processing steps. Gari frying was carried out at 165 °C for 12 min. The dough was prepared by introducing the gari into hot (boiled) water and stirred until a smooth textured dough was achieved.

### 2.3. β-Carotene Extraction and HPLC Analysis

The extraction and instrumentation were carried out using HarvestPlus methods [28] with slight modifications to the sample weight, which varied across each step of processing. Waters HPLC system (Water Corporation, Milford, MA, USA) consisting of a guard-column, C30 YMC Carotenoid column (4.6 × 250 mm, 3 μm) supplied by YMC Korea Co., Ltd., Sungnam-si, Korea, Waters 626 binary HPLC pump, 717 autosampler, and a 2996 photodiode array detector (PDA) was used for β-carotene quantification. Chromatograms were generated at 450 nm (Appendix C) and subsequent identification of cis and trans isomers of β-carotene was done. The modifications to the extraction and the full description of the instrumentation applied were adapted from literature where they have been fully described [29,30].

### 2.4. Dry Matter Content

An oven-drying method was used to determine dry matter content. Samples (fresh cassava roots, intermediate, or final products) were oven-dried for 20 to 24 h at 105 °C until a constant weight was achieved. Weight before and after drying was taken and used to calculate the lost weight and the dry matter content [31].

### 2.5. True Retention Calculation

After adjustments were made for weight and moisture content changes, percentage true retention was calculated as reported [20,32,33]. Refer to Appendix A for sample calculation.

### 2.6. Dietary Intake Assessment

Retrospective dietary intake data from 100 primary school children, 102 female and 100 male in-school adolescents, and 108 adult women were used for the study. The data was obtained from dietary intake assessments which used a multipass 24-h dietary recall method to elicit information from selected respondents. These assessments are periodically carried out by the Department of Human Nutrition of the University of Ibadan, Nigeria, and are always collected under the full guidance and approval of the University’s ethics review committee. The mean portion sizes (in grams) of commonly consumed cassava products (gari and its dough) were extracted from the full dietary survey data and averaged using a spreadsheet.

### 2.7. Estimation of Possible Contribution to Vitamin A Intake

Mean portion size (in grams) of the commonly consumed cassava products from white variety gari and its dough was compared to β-carotene concentrations in the similar products from yellow cassava varieties and was used to calculate possible contribution to Estimated Average Requirement (EAR) for vitamin A intake. The age range of the children whose dietary intake data was considered was 4–8 years. The adolescents ranged from 14 to18 years old and the women were aged between 20 and 50 years. The EAR values were extrapolated from the Dietary Reference Intake Tables [34]. EAR values were 275 µg for children, 630 µg for adolescent males, 485 µg for adolescent females, and 500 µg for women. The bioconversion factor of 12 µg to 1 Retinol Activity Equivalent (RAE) was applied [34]. Refer to Appendix B for sample calculation.

### 2.8. Statistical Analysis

Data of analytical values were expressed as Mean ± Standard deviation (SD). The Statistical interaction between varieties and different processing methods on β-carotene concentrations and corresponding retention of intermediate and final products were evaluated using a linear regression analysis while means separation was analyzed using Duncan’s Multiple range test. The level of significance was set at *p* < 0.05. IBM SPSS Statistics for Windows, version 20 (IBM Corp., Armonk, NY, USA) was used for the statistical analyses.

## 3. Results and Discussion

### 3.1. β-Carotene Concentrations and Retention

The β-carotene (µg/g) concentrations in fresh weight basis (FWB) and their percentage true retention starting from the fresh roots through intermediate to final products (gari and its dough) are presented in Table 1 and Table 2. The concentration in fresh roots ranged from 5.32 µg/g to 7.81 µg/g in TMS 1371 and NR 0220, respectively. For the grated mash, after fermenting for one day, the mean percentage retention for the four varieties was 87.35%, ranging from 76% to 97.7% in TMS 0539 and TMS 0593, respectively. The mean β-carotene concentration of the dewatered mash was 10.94 µg/g. The true retention values further reduced in the dewatered mash with the range being 16.8% to 31.6%. This was the same trend observed until the final products (gari and cooked dough) were obtained. However, there was a substantial decrease in the mean of β-Carotene concentrations of *gari* (16.34 µg/g) and dough (2.89 µg/g). Table 2 shows the β-carotene (µg/g) concentrations and their percentage true retention starting from the fresh roots through intermediate to final products (gari and dough) after the grated mash was fermented for three days. Mean retention after three days fermentation was 86.19%. This ranged from 72 to 93% in TMS 1371 and NR 0220, respectively.

The mean β-carotene concentration of the dewatered mash was 5.12 µg/g, which is slightly higher than that of the fresh roots. The true retention values further reduced in the dewatered mash with the range being 19.2 to 42.7%. The largest decrease in true retention during processing was found in the retention capacities of the varieties from the mash (G2% and G6%) to those from the dewatered mash (G3% and G7%) in one day and three days fermentation, respectively. This could be attributed to a loss of moisture and soluble solids which results in increased concentration of carotenoid content compared to the weights of the cassava in the obtained in the two steps. This observation is consistent with literature findings on retention during cassava processing, where a reduction in moisture content during processing steps resulted in a large drop in the retention of carotenoids [17,18,21,24].

A slight change in the retention range from the dewatered to the sieved mash could also be due to a slight reduction in the weight after sieving of larger size particles. The changes observed in the fried gari are attributed to the exposure to heat during frying which concentrates the cassava granules and conversely causes further loss of β-carotene. Production of gari, which is the most popular product of cassava processing in sub-Saharan Africa has importance when considering the bioefficacy of biofortified cassava consumption in vitamin A deficient populations since extended roasting could result in higher carotenoid loss [19]. This study optimized the roasting process by frying for 12 min as suggested in the literature [35]. Further studies on more varieties may be needed to ascertain how newer varieties may behave at different frying temperatures since genotypes vary in retention ability during processing [36]. Similar to products from one-day fermentation batch, there was a sharp decrease in the mean of β-carotene concentrations of gari and its dough (from 3-day fermentation), which reduced from 14.52 µg/g and 6.13 µg/g.

There was an observed decrease in the retention of β-carotene from the beginning of processing to the final products (gari and its dough), irrespective of fermentation periods of one day or three days. This is explained by the degradation of β-carotene during processing [37]. It has also been reported that processing of yellow-fleshed cassava into consumable products can result in major or minor losses of carotenoids through the interactions of physical factors, like heat, light, oxygen, food enzymes, or a combination of all [16,17,18]. These observed losses could also be due to carotenoids isomerization and oxidation which is the breakdown of trans-carotenoids to their cis isomers due to increased contact with moisture, heat treatment, and exposure to light [38,39]. More recent findings confirm these depletion patterns in Cassava products consumed in Sub-Saharan Africa [21,40].

Despite the similarity in the trend of β-carotene loss, there are some marked differences in the retention and concentration values obtained from fermentation for one day and fermentation for three days as probed in this study. They include the slightly lower retention values of the mash fermented for three days (G6) over the mash fermented for one day (G2). This slight reduction in the percentage true retention is consistent with literature which establishes a lowered percentage true retention of carotenoids with longer fermentation [29,41]. Another peculiar observation was the major reduction in percentage true retention and concentration when gari is cooked into its dough. This decrease in retention is observed between gari and its dough, where retention values reduce to less than 10%, except for 1371 fermented for three days that showed 16.57%, thus signifying a critical loss in the β-carotene content in the dough produced from gari. This loss may be due to the depletion in carotenoid content after using hot water to make the dough where the gari is introduced into hot water and stirred continuously until the dough has a smooth texture. Another explanation could be the increased moisture content (in eba) which affects dry matter.

### 3.2. Statistical Interaction between Varieties and Processing Methods

Table 3 shows the statistical interaction of varieties and the different fermentation methods on β-carotene concentrations (µg/g) and the percentage true retention in intermediate and final products. Each stage behaves independently of the other except for the concentrations of grated mash and the percentage retention of sieved cassava mash. There is no significance in the interactive effect of methods on β-carotene concentration at the fermentation stage. While longer fermentation had a minimal effect on percentage true retention values across the processing steps, it had a significant statistical effect on the concentration of β-carotene in all intermediate products and final products—gari and dough. The results of statistical comparisons show that the factors affecting the retention of carotenoids in gari and its dough during processing are not singular. There is dependence on not only the processing method but also the variety. These effects are important (especially the change in fermentation period) considering the common practice of gari production that involves at least three days of fermentation of the grated mash [25,26]. Even though fermenting for a day resulted in comparative advantage, the difference in sensory qualities may be questionable. The non-significance of retention in the mash from the two fermentation plans is expected since the fermentation of the two batches started at the same time.

### 3.3. Dietary Intake and Possible Nutrient Intake

The Nigerian Food Consumption survey [42] reported a high consumption frequency for Cassava food products, thus providing a basis for comparisons with the portion sizes of products from the already existing white variety. The average portion size distribution as presented in Table 4 shows the comparisons of the dietary intake of children, adolescents, and women in southwestern Nigeria. The consumption of cassava products in the sampled respondents shows that gari had the least mean portion size across the three age groups—children (116 g), adolescents (120 g and 119 g), and women (87 g)—when compared with the cooked dough: children (236 g), adolescents (352 g and 345 g), and women (598 g).

The results also show that the dough is consumed more in terms of portion size compared to gari across all groups surveyed. Estimation of possible contributions of biofortified gari and its dough to the estimated average requirement of vitamin A in children, adolescents, and women assumes similar portion sizes will be consumed if the respondents were served the new products.

Variety NR 0220 gave the highest contribution from gari across all age groups ranging from 13.7 to 33.2% in women and children respectively. While variety NR 0220 was the second highest contributor to vitamin A intake, variety TMS 1371 made into eba is estimated to contribute highest to nutrient intake. In adolescent boys it could provide 17.6%, while for women it could provide 37% of EAR of vitamin A. Comparatively, the contribution of eba was lower in all age groups and varieties considered in this study when compared to the contribution of gari. The exceptions were variety TMS 1371 for adolescent males and women. Considering the β-carotene levels in gari (µg/g) presented in Table 1 and Table 2, the estimated contribution of gari was expectedly higher than that of the dough for all age categories. This suggests that if gari is consumed frequently, it may better contribute to vitamin A intake compared to eba. The physical nature of gari is a major advantage and this could make it a useful vehicle since it is dry and contains more nutrients per weight when compared to the dough which has a lesser dry matter per weight. The lowest portion size for gari consumption was observed with women showing that it may not be the best vehicle for improving vitamin A intake in women. Although the β-carotene levels decline significantly during cooking gari into the dough, the remainder of carotenoids in the dough has the possibility of contributing to pro-Vitamin A intake [43]. This is full comparisons are described in Table 5.

Even though the consumption of the dough is higher in weight across all age groups, the impact of the drop in retention and concentration when cooking gari into its dough is noticeable in Table 5 with most of the varieties considered in this study. From these observations, the concentrated nature of gari per unit weight confirms that it may be a more viable vehicle of dietary pro-Vitamin A content than its dough. This, therefore, implies that extensive processing may be a hindrance in the utilization of these new crops. High depletion of β-carotene levels after cooking was similarly observed from reported literature [40]. Even though the impact of consuming gari and its dough on vitamin A serum concentrations is not yet fully established in VAD populations, the results presented give a hint that the newly released varieties of Cassava have a chance of reducing the burden of VAD in sub-Saharan populations where it is still endemic. Scaling up the adoption and utilization of these new varieties will reduce the VAD burden. Another point worthy of note is that since β-Carotene values had to be converted to retinol activity equivalents before estimation of Vitamin A intake, it should also be noted that the higher theoretical bioconversion ratio of 12 µg:1 RAE [34] as against a lower ratio of about 4.5 µg:1 RAE reported in a bioavailability study of Biofortified cassava porridges [44] could have resulted in the low estimates presented for vitamin A intake in this study. This uncertainty supports an urgent need for scale-up and assessment of the impact of introducing these products in sub-Saharan Africa.

From a recent survey of factors affecting the adoption of cassava varieties [45], numerous determinants could influence the adoption of new cassava varieties and result in the farmers’ favoritism for agronomic and economic qualities above nutritional information. However, these challenges should not deter dissemination efforts since, in the local diet, the dough is usually commonly consumed with soups and stews which have substantial carotenoids content [46]. This combination as obtained in a meal could contribute to increased micronutrient intake which compensates for the depleted β-carotene content.

## 4. Conclusions

Biofortification of cassava varieties presents a viable and promising intervention for tackling vitamin A deficiencies in disease-burdened populations of sub-Saharan Africa. This study provides evidence that retention of β-carotene in biofortified cassava is not only dependent on genotype, but also on the processing method. While this study proves that short fermentation can result in improved retention of β-carotene content, further studies may be needed to ascertain the effect of a short fermentation period on the organoleptic properties of gari and its dough since increased time of fermentation has been established to increase the desired sourness in gari made from the white variety [47]. This study also highlights a challenge in providing substantial pro-vitamin A content across age groups when considering locally practiced processing methods, which result in products with lowered retention. This can, however, be managed by nutrition education targeted at improving dietary diversity. Also, since further breeding of varieties with higher β-carotene content is ongoing, it is expected that these efforts can provide varieties with higher pVA content which will result in an increased contribution of pro-vitamin A to usual nutrient intake. It is anticipated that the information presented will be useful when the questions of bioavailability and bioefficacy after consumption of these popular cassava products are raised.

## Figures and Tables

**Table 1:** β-carotene (µg/g) concentrations (fresh weight basis) and their percentage true retention in cassava products from yellow cassava varieties (one-day fermentation) ^1^.

Varieties	Fresh Roots	G2	G2 (%)	G3	G3 (%)	G4	G4 (%)	G5	G5 (%)	G10	G10 (%)
**0593**	6.75 ± 0.07 ^b^	6.60 ± 0.14 ^c^	97.7 ± 0.42 ^d^	10.60 ± 0.57 ^a^	49.5 ± 0.71 ^c^	10.38 ± 0.17 ^c^	31.6 ± 0.57 ^c^	13.50 ± 0.0 ^a^	29.3 ± 0.0 ^c^	3.33 ± 0.0 ^c^	7.2 ± 0.0 ^c^
**0539**	6.96 ± 0.06 ^b^	4.03 ± 0.0 ^a^	76 ± 0.0 ^a^	11.15 ± 0.2 ^a^	50.3 ± 0.42 ^c^	6.38 ± 0.14 ^a^	22.3 ± 0.42 ^b^	17.66 ± 0.0 ^c^	20.8 ± 0.0 ^b^	1.94 ± 0.0 ^a^	2.1 ± 0.0 ^a^
**0220**	7.81 ± 0.13 ^c^	9.10 ± 0.14 ^d^	94 ± 1.41 ^c^	10.61 ± 0.0 ^a^	32.5 ± 0.0 ^a^	14.86 ± 0.0 ^d^	22.3 ± 0.0 ^b^	18.37 ± 0.18 ^d^	14.5 ± 0.71 ^a^	2.68 ± 0.03 ^b^	1.26 ± 0.34 ^a^
**1371**	5.32 ± 0.03 ^a^	5.20 ± 0.0 ^b^	81.7 ± 0.0 ^b^	11.39 ± 0.0 ^a^	35.4 ± 0.0 ^b^	7.61 ± 0.0 ^b^	16.8 ± 0.0 ^a^	15.83 ± 0.17 ^b^	14.4 ± 0.3 ^a^	3.60 ± 0.07 ^d^	4.3 ± 1.41 ^b^
**Mean**	6.71 ± 0.96	6.23 ± 2.02	87.35 ± 9.46	10.94 ± 0.43	41.93 ± 8.61	9.81 ± 3.48	23.25 ± 5.69	16.34 ± 2.01	19.73 ± 6.52	2.89 ± 0.69	3.72 ± 2.52
**S.E**	0.52	1.09	5.10	0.20	4.65	1.88	3.07	1.09	3.53	0.37	1.33
**C.V (%)**	15.4	35.0	11.7	3.7	22.2	38.4	26.4	13.3	35.8	25.7	71.4

G2—Grated Mash; G3—Dewatered mash; G4—Sieved mash; G5—Gari; G10—Cooked Dough. ^1^ Mean of triplicate determination. Means with different letters along columns are significantly different at *p* < 0.05.

**Table 2:** β-Carotene (µg/g) concentrations (fresh weight basis) and their percentage true retention of cassava products from Yellow Cassava varieties (three-day fermentation) ^1.^

Varieties	Fresh Roots	G6	G6 (%)	G7	G7 (%)	G8	G8 (%)	G9	G9 (%)	G11	G11 (%)
**0593**	6.75 ± 0.07 ^b^	6.80 ± 0.07 ^bc^	92.97 ± 0.04 ^c^	3.89 ± 0.16 ^a^	21 ± 0.71 ^b^	3.66 ± 0.08 ^a^	13.04 ± 0.1 ^b^	7.19 ± 0.0 ^a^	10 ± 0.0 ^a^	1.52 ± 0.28 ^a^	5.4 ± 0.57 ^c^
**0539**	6.96 ± 0.06 ^b^	6.20 ± 0.28 ^b^	86.8 ± 0.14 ^b^	5.72 ± 0.00 ^b^	19.2 ± 0.0 ^a^	4.48 ± 0.0 ^b^	11.2 ± 0.0 ^a^	7.55 ± 0.57 ^ab^	10.39 ± 2.8 ^a^	1.72 ± 0.0 ^a^	0.05 ± 0.0 ^a^
**0220**	7.81 ± 0.13 ^c^	8.02 ± 0.0 ^c^	93 ± 0.0 ^c^	6.35 ± 0.00 ^b^	38.4 ± 0.0 ^c^	6.35 ± 0.0 ^c^	26.4 ± 0.0 ^c^	9.44 ± 0.31 ^c^	16 ± 2.83 ^b^	2.20 ± 0.4 ^a^	2.5 ± 0.71 ^b^
**1371**	5.32 ± 0.03 ^a^	5.1 ± 0.71 ^a^	72 ± 1.13 ^a^	4.51 ± 0.47 ^a^	42.7 ± 0.42 ^d^	6.28 ± 0.31 ^c^	41.85 ± 0.2 ^d^	8.37 ± 0.0 ^b^	21.7 ± 0.0 ^c^	3.79 ± 0.16 ^b^	16.57 ± 0.3 ^d^
**Mean**	6.71 ± 0.96	6.50 ± 1.02	86.19 ± 9.18	5.12 ± 1.05	30.33 ± 11.1	5.19 ± 1.25	23.12 ± 13.2	8.14 ± 0.96	14.52	2.31 ± 0.97	6.13
**S.E**	0.52	0.62	4.95	0.56	5.98	0.67	7.10	0.50	2.76	0.51	3.65
**C.V (%)**	15.4	19.2	11.5	21.9	39.4	25.8	61.4	12.3	37.9	44.5	119.0

G6—Grated Mash; G7—Dewatered mash; G8—Sieved mash; G9—Gari; G11—Cooked Dough. ^1^ Mean of triplicate determination. Means with different letters along columns are significantly different at *p* < 0.05.

**Table 3:** Mean squares of statistical interaction of variety and different processing steps on β-carotene concentration and retention.

Products	Mash	% TR	Dewatered Mash	% TR	Sieved Mash	%TR	Fried Garri	%TR	Cooked Dough	%TR
**P variety**	9.34 ***	327.86 ***	1.32 ***	15.56 ***	19.29 ***	0.065 ^ns^	8.68 ***	17.45 ***	2.46 ***	75.13 ***
**P method**	0.08 ^ns^	5.36 ***	135.59 ***	538.24 ***	85.19 ***	108.29 ***	269.95 ***	109.31 ***	1.35 ***	23.33 ***
P variety*method	2.423 ***	76.25 ***	1.51 ***	443.11 ***	12.59 ***	370.64 ***	2.74 ***	142.36 **	0.75 ***	45.40 ***

** = Significance at *p* < 0.01, *** = Significance at *p* < 0.001, ns = not significant. % TR = percentage true retention.

**Table 4:** Mean with standard deviation (in grams) of common cassava products (gari and eba) consumed by children, adolescents, and women.

Product	Children	Adolescents (Male)	Adolescents (Female)	Women
***Gari*** **(grams)**	116 ± 30.4	120.4 ± 46.7	119.4 ± 46.7	87 ± 24.9
***Eba*** **(grams)**	236 ± 106.6	352 ± 120.7	345.1 ± 120.7	598 ± 259.3

**Table 5:** Estimated percentage contributions of cassava products to estimated average requirement (EAR) of vitamin A.

	Variety	*Gari (%)	*Eba* (%)
**Children**	TMS 0593	25.3	10.8
	TMS 0539	26.5	12.3
	NR 0220	33.2	15.7
	TMS 1371	29.4	27.1
**Adolescents (Male)**	TMS 0593	11.5	7.1
	TMS 0539	12.1	8.0
	NR 0220	15.0	10.2
	TMS 1371	13.3	17.7
**Adolescents (Female)**	TMS 0593	14.8	9.0
	TMS 0539	15.5	10.2
	NR 0220	19.4	13.0
	TMS 1371	17.2	22.5
**Women**	TMS 0593	10.4	15.2
	TMS 0539	11.0	17.1
	NR 0220	13.7	21.9
	TMS 1371	12.1	37.8

* = Gari processed from three-day fermentation.

**Figure A1:**
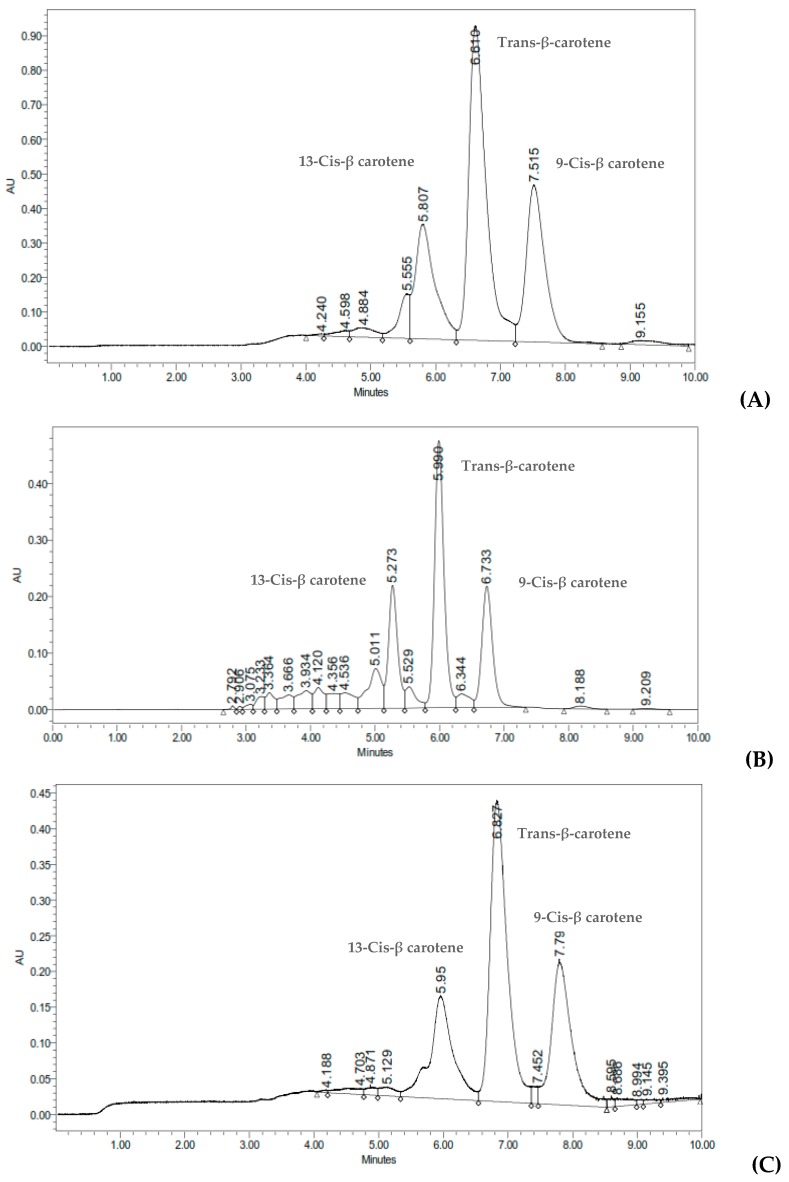

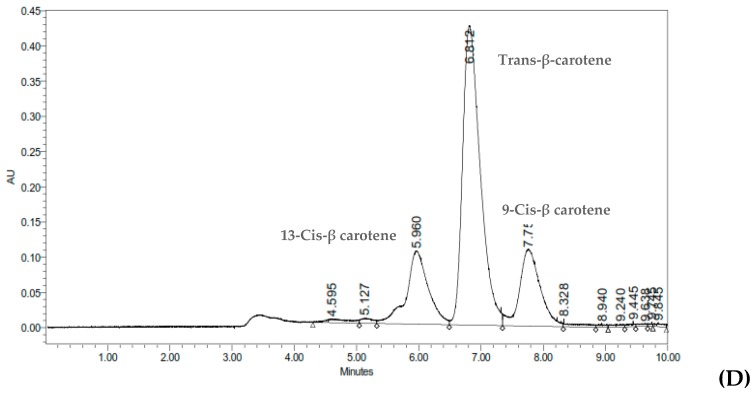
Showing chromatograms of isomers of beta-carotene from fresh cassava roots of (**A**) TMS 0593, (**B**) TMS 0539, (**C**) NR 0220, and (**D**) TMS 1371.

## Appendix A

Calculation of % true retention according to Murphy et al. (1975)

(1) $$\frac{\mathrm{Nutrient}\;\mathrm{content}\;\mathrm{per}\;g\;\mathrm{of}\;\mathrm{processed}\;\mathrm{food}\;\times \;\mathrm{weight}\;\mathrm{after}\;\mathrm{processing}\;\times 100\;}{\mathrm{Nutrient}\;\mathrm{content}\;\mathrm{per}\;g\;\mathrm{of}\;\mathrm{raw}\;\mathrm{food}\;\times \;\mathrm{weight}\;\mathrm{of}\;\mathrm{food}\;\mathrm{before}\;\mathrm{processing}}$$

The weight was then adjusted for moisture content (dry matter)

Example

Calculation of % TR of G6 (gari mash of three days) for Variety 1371 in Table 2

β-Carotene of raw = 212 µg/g	β-Carotene of G6 mash = 198 µg/g
Weight of raw = 600 g	Weight of G6 mash = 560 g
Dry matter of raw = 28%	Dry matter of G6 mash = 23%

(2) $$\%\mathrm{TR}\;=\;\frac{560\;\times 0.23\;\times \;198}{600\;\times 0.28\;\times \;212}\;\times \;\frac{100}{1}\;=\;71.60\%$$

## Appendix B

2. Calculation of contribution of gari to EAR of vitamin A in Children (Table 4)

Variety 1371

EAR for Children (4–8 years) = 275 µg/day

Mean Portion Size = 116 g

1 g = 8.37 µg (From G9 on Table 2) 116 g = 970.92 µg

RAE (Retinol Activity Equivalents) using a bioconversion of 12 µg to 1 RAE

(3) $$\mathrm{RAE}\;=\;\frac{970.92}{12}\;=\;80.91\;g$$

(4) $$\%\;\mathrm{EAR}\;=\;\frac{80.91}{275}\;\times \;100\;=\;29.42\%$$
